# Belladonna in Hooping-Cough

**Published:** 1845-05

**Authors:** 


*Belladonna in Hooping-Cough.*—Dr. Waller had of late tried belladonna in two cases of hooping-cough with the best results. He gave the extract in a twelfth of a grain dose, three times a day, to a child four years of age. In these cases there was simply the spasmodic cough, and no indication whatever of the presence of inflammation. Prussic acid and conium had failed in affording any permanent or marked relief.

Mr. Crisp had in the majority of cases found the antiphlogistic plan of treatment in the early stages of hooping-cough to be the best treatment. In robust and healthy children the application of leeches, followed by blisters, and the use of tartar emetic, had usually been attended by the best results. He viewed the disease generally as inflammatory, or at all events congestive. Contrasting the antiphlogistic treatment with that which he had formerly pursued, he had every reason to be satisfied with the change that he had made. With respect to the use of prussic acid, he had usually found it of service only for a day or two.

Dr. Wiltshire, at the Infirmary for Children, had treated simple uncomplicated hooping-cough with two or three emetics of ipecacuanha, one every morning or second morning, followed for two or three days with nauseating doses of antimony, and had found this plan of great service in the early stages of hooping-cough. Hemlock and ipecacuanha were of benefit afterwards in relieving the cough. With respect to belladonna, he was fearful of its employment, for notwithstanding the evidence in its favor, adduced by certain German practitioners, there were others who had found that this medicine has a tendency to increase vascular action in the brain, and to the production of hydrocephalus.

Dr. Chowne, in the treatment of hooping-cough, laid particular stress on the necessity of keeping the patient in a warm temperature, and using every means to prevent his catching cold. Nauseating doses of ipecacuanha were frequently of benefit. With reference to the production of emphysema by hooping-cough he did not believe it possible.

Dr. Clutterbuck looked upon hooping-cough as a specific disease, produced by a specific cause—it was an inflammation of the bronchial membrane of a specific character, but apt to induce inflammation of other organs, such as of the lungs or of the head, and it was these complications that constituted the dangerous characters of hooping-cough. He had little confidence in any remedy for this affection. The disease should be narrowly watched, with the view of prevention, rather than of active treatment; if mild, nothing was necessary to be done, it would terminate spontaneously; if threatened the complications he had alluded to decided antipholgistic means were demanded.

Dr. Golding Bird regarded the hoping-cough, in the first stage, as invariably inflammation of the lining membrane of the bronchial tubes, larynx, and trachea, of a specific character, and implicating, in some manner, the par vagum. This inflammation lasted a definite period, which was influenced by constitution, and other causes. In the second stage of the affection, the disease was *nervous*, the specific irritation of the par vagum being kept up, altogether independent of inflammation; or, if this were present, it was accidental; the hooping was afterwards protracted, by the influence of habit. In the first stage, remedies for bronchitis were advisable—such as emetics, diaphoretics, the warm bath, and a warm temperature. When the inflammotory stage was passed, the object of the practitioner was to subdue irritation in the par vagum, and this was effected by the agency of narcotics—such as conium, in conjunction with the carbonate of potash, hemlock, and hydrocyanic acid. Embrocations to the spine and chest were also useful. When bronchorrhœa became troublesome, small doses of alum, with sedatives, were employed with advantage. When the bronchorrhæa had ceased, tonics were indicated,—the kind of tonic to be determined by the constitution of the patient. Emphysema did not occur as the consequence of hooping-cough.

The President had found, in some cases where belladonna was given, that the poisonous, rather than the curative effects of that remedy developed themselves, even though the doses administered were remarkably small. With reference to the irritation of the par vagum in hooping-cough, he related a case in which this nerve had become exposed, from the formation of an abscess, or other cause; and it was remarkable, that when the nerve was in contact with the air, a spasmodic resembling hooping-cough was produced: when the nerve was covered over by a cicatrix, the hooping-cough ceased.

